# Evaluation of sex differences in dietary behaviours and their relationship with cardiovascular risk factors: a cross-sectional study of nationally representative surveys in seven low- and middle-income countries

**DOI:** 10.1186/s12937-019-0517-4

**Published:** 2020-01-13

**Authors:** Briar L. McKenzie, Joseph Alvin Santos, Pascal Geldsetzer, Justine Davies, Jennifer Manne-Goehler, Mongal Singh Gurung, Lela Sturua, Gladwell Gathecha, Krishna K. Aryal, Lindiwe Tsabedze, Glennis Andall-Brereton, Till Bärnighausen, Rifat Atun, Sebastian Vollmer, Mark Woodward, Lindsay M. Jaacks, Jacqui Webster

**Affiliations:** 1 The George Institute for Global Health, University of New South Wales, Sydney, Australia; 2 Division of Primary Care and Population Health, Department of Medicine, Stanford University, Stanford, USA; 3 Institute of Applied Health Research, University of Birmingham, Birmingham, UK; 4 Medical Research Council/Wits University Rural Public Health and Health Transitions Research Unit, Faculty of Health Sciences, School of Public Health, University of the Witwatersrand, Johannesburg, South Africa; 5 Massachusetts General Hospital, Boston, USA; 6 Health Research and Epidemiology Unit, Ministry of Health, Thimphu, Bhutan; 7 Non-Communicable Disease Department, National Center for Disease Control and Public Health, Petre Shotadze Tbilisi Medical Academy, Tbilisi, Georgia; 8 Division of Non-Communicable Diseases, Kenya Ministry of Health, Nairobi, Kenya; 9 DFID/NHSP3/MEOR, Abt Associates, Lalitpur, Nepal; 10 Ministry of Health, Mbabane, Eswatini; 11 Non-Communicable Diseases, Caribbean Public Health Agency, Port of Spain, Trinidad and Tobago; 12 Department of Global Health and Population, Harvard T.H. Chan School of Public Health, Boston, USA; 13 Heidelberg Institute of Global Health (HIGH), Faculty of Medicine and University Hospital, Heidelberg University, Heidelberg, Germany; 14 Africa Health Research Institute (AHRI), Somkhele an Durban, Durban, South Africa; 15 Department of Economics and Centre for Modern Indian Studies, University of Göttingen, Göttingen, Germany; 16 The George Institute for Global Health, University of Oxford, Oxford, UK; 17 Department of Epidemiology, Johns Hopkins University, Baltimore, USA

**Keywords:** Salt use behaviour, Fruit, Vegetables, Sex differences, Hypertension, Diabetes, Waist circumference, Africa, Asia, Americas

## Abstract

**Background:**

Cardiovascular diseases (CVD) are the leading causes of death for men and women in low-and-middle income countries (LMIC). The nutrition transition to diets high in salt, fat and sugar and low in fruit and vegetables, in parallel with increasing prevalence of diet-related CVD risk factors in LMICs, identifies the need for urgent action to reverse this trend. To aid identification of the most effective interventions it is crucial to understand whether there are sex differences in dietary behaviours related to CVD risk.

**Methods:**

From a dataset of 46 nationally representative surveys, we included data from seven countries that had recorded the same dietary behaviour measurements in adults; Bhutan, Eswatini, Georgia, Guyana, Kenya, Nepal and St Vincent and the Grenadines (2013–2017). Three dietary behaviours were investigated: positive salt use behaviour (SUB), meeting fruit and vegetable (F&V) recommendations and use of vegetable oil rather than animal fats in cooking. Generalized linear models were used to investigate the association between dietary behaviours and waist circumference (WC) and undiagnosed and diagnosed hypertension and diabetes. Interaction terms between sex and dietary behaviour were added to test for sex differences.

**Results:**

Twenty-four thousand three hundred thirty-two participants were included. More females than males reported positive SUB (31.3 vs. 27.2% *p*-value < 0.001), yet less met F&V recommendations (13.2 vs. 14.8%, *p*-value< 0.05). The prevalence of reporting all three dietary behaviours in a positive manner was 2.7%, varying by country, but not sex. Poor SUB was associated with a higher prevalence of undiagnosed hypertension for females (13.1% vs. 9.9%, *p*-value = 0.04), and a higher prevalence of undiagnosed diabetes for males (2.4% vs. 1.5%, *p*-value = 0.02). Meeting F&V recommendations was associated with a higher prevalence of high WC (24.4% vs 22.6%, *p*-value = 0.01), but was not associated with undiagnosed or diagnosed hypertension or diabetes.

**Conclusion:**

Interventions to increase F&V intake and positive SUBs in the included countries are urgently needed. Dietary behaviours were not notably different between sexes. However, our findings were limited by the small proportion of the population reporting positive dietary behaviours, and further research is required to understand whether associations with CVD risk factors and interactions by sex would change as the prevalence of positive behaviours increases.

## Background

Cardiovascular diseases (CVD) are the leading causes of death for men and women in low- and middle-income countries [1, 2]. Current evidence suggests that this burden is partly the result of a rapid nutrition transition [3–5], and consequent increases in cardiovascular risk factors, including obesity [6], diabetes [7], and hypertension [8]. Earlier systematic reviews and prospective cohort studies have provided evidence of the effect of dietary factors, such as high salt intake [9, 10], low consumption of fruits and vegetables [11–14], and the increased consumption of trans- and saturated fat in place of mono- and poly-unsaturated fat [12, 13, 15–17] on increased cardiovascular risk.

The weight of the evidence demonstrating the burden of ill health due to diets high in salt [9, 10], low in fruits and vegetables [11–14], and high in trans- and saturated fats [12, 13, 15–17] has enabled the development of global targets and recommendations by the World Health Organization (WHO) to reduce dietary risks for CVD, and non-communicable diseases (NCD) more broadly. The WHO Global NCD Action Plan [18] specifies targets to reduce population salt intake by 30%, and for adults to consume at least 400 g of fruit and vegetables a day (approximately five servings a day). There are also global targets to eliminate the use of trans-fats [19] and a recommendation to reduce the intake of saturated fats, aiming for intake to be 10% or less of total energy intake [20]. In order to monitor population-level NCD risk factors, including dietary behaviours, the WHO has supported the implementation of national surveys called the “STEPwise approach to surveillance” or “STEPS” [21]. These surveys contain questions on dietary behaviours such as salt use, fruit and vegetable consumption, and type of fat and oil used in cooking. Analysis of these surveys can inform country-specific strategies for reducing NCD risk, on reduction of dietary risk.

In the past decade a growing body of high-quality research has identified differing impacts of non-dietary cardiovascular risk factors, such as high systolic blood pressure, diabetes and smoking, on disease outcomes for men and women [22, 23]. There is evidence from studies conducted in high income countries that self-reported dietary behaviours differ for men and women [24, 25]. However, there is a dearth of similar research from low-and-middle income countries, and on potential differences in the association between dietary behaviours and disease outcomes by sex. Given the Sustainable Development Goals (SDG) of achieving good health and well-being (SDG 3) and gender equality (SDG 5) [26], it is important to investigate sex differences in dietary behaviours and any relationship with health outcomes in a global setting to inform nutrition interventions and thereby reduce the burden of CVD and its adverse financial consequences [27].

The objectives of this study were to use individual-level data from nationally representative surveys to investigate sex differences in (1) the dietary behaviours of salt use, fruit and vegetable consumption and type of oil and fat used in cooking, and (2) the association of these behaviours with the prevalence of three key CVD risk factors: high waist circumference, hypertension and diabetes. Given the hypothesis that disease diagnosis may change behaviour, and therefore those with diagnosed disease may be more likely to report more positive dietary behaviours [28], investigation of associations with both undiagnosed and diagnosed hypertension and diabetes were conducted.

## Methods

### Data sources

This study utilised data from nationally representative surveys conducted in Bhutan, Eswatini, Georgia, Guyana, Kenya, Nepal and St Vincent and the Grenadines; all upper-middle, lower-middle, or low-income countries [29] at the time the surveys were conducted. The method of data acquisition and pooling have previously been described [30–32]. In brief World Health Organization (WHO) Stepwise Approach to Surveillance (STEPS) surveys [33] conducted in low, low- middle, or upper-middle income countries since 2005 were searched for. The search was limited to surveys conducted since 2005, as these studies were considered contemporary enough to be included in the same analysis. WHO STEPS surveys use a standardised questionnaire and protocol to monitor non-communicable disease risk at a population level, with the questionnaire comprising three steps: step one “behavioural measurements”, step two “physical measurements” and step three “biochemical measurements” [21, 33, 34]. Survey contacts were approached for the de-identified individual level data to be pooled for analyses. Data was pooled if signed agreement was made and they had a response rate ≥ 50%; participants were aged 15 years or older; included data on waist circumference, and/or a biomarker for diabetes (either a glucose measurement or HbA1c), and/or a measurement of blood pressure. For the current analyses surveys were included if questions on salt behaviour, fruit and vegetable intake, and the use of fats and oils for cooking were asked, seven out of 46 surveys. The surveys used a two-stage cluster random sampling design, with one person from each household (within the defined age range) randomly selected to complete the survey. All surveys were carried out by a trained data collection team member in the household setting, or at a conveniently-located health center and data on the three questionnaire steps were collected during the same visit.

### Terminology – sex - gender

A person’s sex is recorded in the WHO STEPS surveys by the interviewer documenting the observed sex of the participant (binary, male or female) [21]. While acknowledging that the self-report of dietary behaviours is likely to be influenced by a person’s identity and social constructs, and therefore also related to a person’s gender, to be in line with the data collected, the term “sex”, and corresponding terms “male” and “female”, are used throughout this paper [35].

### Classification of dietary behaviours

Diet behaviours [36] of salt use, fruit and vegetable consumption and type of oil and fat used in cooking are included within “Step 1 – Behavioural Measurements” of the questionnaire, and are the only dietary behaviour variables included in STEPS [21].

#### Salt use behaviours

There are seven salt use behaviour questions included in STEPS [21]: 1. *How often do you add salt or salty sauce such as soy sauce to your food right before you eat it or as you are eating it?* 2. *How often is salt, salty seasoning or a salty sauce added in cooking or preparing foods in your household? Do you do any of the following on a regular basis to control your salt intake:* 3. *Limit consumption of processed foods?* 4. *Look at the salt or sodium content on food labels?* 5. *Buy low salt/sodium alternatives?* 6. *Use spices other than salt when cooking?* 7. *Avoid eating foods prepared outside of a home?* The first two questions used a 5-point Likert response scale with options of: always, often, sometimes, rarely, or never. These answers were assigned a value of 0, 0.25, 0.5, 0.75 or 1, respectively. The other five questions used a “yes” or “no” response, which was assigned a value of 1 and 0, respectively. To investigate the prevalence of positive (good) compared to poor salt used behaviour, the response values for all the seven questions were summed, and individuals with a score of 0.5 (50%) or greater were labelled as having positive (good) salt use behaviour. Another method of scoring salt use behaviour and categorising into positive vs. poor behaviour was not identified in the literature, and therefore other options of quantification were tested. These included an ordinal 4-point score (categorising into of 25, 50, 75 and 100% of the salt behaviour questions answered positively) and a 7-point score (“1” being one question answered positively, through to “7”, being all questions answered positively). Given the low prevalence of positive salt use behaviour the 50% cut-off was used in the main analyses, with the 4-point score and 7-point score used in sensitivity analyses for the association of salt use behaviour with undiagnosed hypertension.

#### Fruit and vegetable intake

In the surveys, participants were asked to report the number of days per week they consume fruits and vegetables. If participants reported that they consumed fruits or vegetables on one or more days a week, they were then asked to state on any given day how many portions of fruits and vegetables they consume. To aid their response, they were shown pictures of local fruits and vegetables to refer to as a portion, corresponding to approximately 80 g. Fruit and vegetable intake (per day) was then calculated using the methods of Frank S et al. [31]. Briefly, individuals were categorised as meeting, or not meeting, the fruit and vegetable recommendations, based on the WHO- recommendation of five 80 g portions of fruit and vegetables, or more, on a given day, equivalent to 400 g or more a day [18].

#### Oil and fat use

Participants were asked to pick the main oil or fat used to prepare meals in their home. Options, specific to the types of oils and fats used in each country, were provided to the participant. Responses were categorized as: vegetable, animal, other, none in particular, or none used. For analysis, this was further collapsed into vegetable oil, all other oils and fats, and no fat or oil used, given the small number of individuals who reported using other types of fats and oils or no use of fats or oils. “Vegetable oil” was used as the reference (or “positive behaviour”) category, based on evidence that suggests plant-based oils are protective for heart health [13, 17].

### Classification of cardiovascular risk factors

#### Waist circumference

Waist circumference in each survey was conducted following the STEPS data collection manual [37]. Data collectors used constant tension tape to measure waist circumference directly against the participant’s skin where possible, or over light clothing if direct contact was not possible. Measurement was taken with a participant in a standing position, with arms relaxed at their sides and at the end of a normal expiration. The point of measurement was the midpoint between the lower section of the last palpable rib and the top of the hip bone. Waist circumference was then recorded to the nearest 0.1 cm, and only one measurement per participant was recorded. Participants were classified as having a “high waist circumference” if their measured value was ≥102 cm for males and ≥ 88 cm for females [38].

#### Hypertension

Detailed country-specific methods of blood pressure measurement are described elsewhere [32]. Briefly, the included surveys followed the STEPS data collection manual [37], which specifies measures to be conducted using digital, automated upper arm monitors, following 15 min of rest. The majority of participants had three blood pressure readings taken, with 3 min rest between each measure. The average of the last two readings were then taken. For individuals with only two measures, the mean of both available measurements was taken; for individuals with only one measure that measure was taken. A person was classified as having hypertension if their average systolic blood pressure (SBP) measurement was greater than 140 mmHg, or their average diastolic blood pressure (DBP) measurement was greater than 90 mmHg, or they reported taking medication for hypertension. We defined a categorical variable of non-hypertensives (reference), undiagnosed hypertension, and diagnosed hypertension. Individuals with self-reported diagnosed hypertension were those who met the criteria for hypertension and also reported a diagnosis of hypertension. Undiagnosed individuals were those who had a high SBP (> 140 mmHg) or a high DBP (> 90 mmHg), did not report taking hypertension medication, and did not report a hypertension diagnosis.

#### Diabetes

Detailed country-specific methods of diabetes measurement are described elsewhere [30]. Briefly, point-of-care fasting capillary glucose measurement was the diabetes biomarker in all surveys apart from the survey conducted in Nepal, where laboratory-based assessment of fasting plasma glucose was used. For the six countries that measured capillary glucose, plasma equivalents were provided. Individuals were asked if they fasted or not prior to the measurement, for those who reported not fasting their blood glucose level was interpreted as a random blood glucose measure. Diabetes was defined as having an average fasting blood glucose (FBG) level of 7 mmol/L or greater, or having a random blood glucose (RBG) level of 11.1 mmol/L or greater, or on medication for diabetes. We evaluated a categorical variable of non-diabetics (reference), undiagnosed diabetes, and diagnosed diabetes. Individuals with self-reported diagnosed diabetes were those who met the criteria for diabetes and also reported a diagnosis of diabetes. Undiagnosed individuals were those who had a high FBG (> 7 mmol/L) or a high RBG (> 11.1 mmol/L), did not report taking diabetes medication, and did not report a diabetes diagnosis.

### Sociodemographic and behavioural variables

Sociodemographic and behavioural factors of interest were sex, age, education, working status, physical activity levels, alcohol use and tobacco use [21].

#### Sociodemographic variables

Age was defined based on the dates of an individual’s birth and the survey, or self-reported age. Age was then categorised into 10-year categories: 15–24, 25–34, 35–44, 45–54, 55–64 and 65 or older. For education a range of options were given including: no formal schooling, less than primary school, primary school completed, secondary school completed, high school completed, college/university completed and post graduate degree. For analysis, education was categorised into “no formal schooling/education”, “primary school attendance only” and “secondary schooling or above”. For working status, a range of occupations were reported including: government employee, non-government employee, self-employed, non-paid, student, homemaker, retired, and unemployed. Of these we classified the self-report of any paid occupation as “working” and any unpaid occupation (for example homemaker) as “not working”.

#### Behavioural variables

STEPS surveys include physical activity questions, covering physical activity at work, for transport and for recreation. For physical activity at work or for recreation, participants were asked if they participate in vigorous or moderate intensity activity, on how many days during the week, and for how long. For transport participants were asked if they walk or cycle for at least 10 min at a time to get to/from places. If they answered “yes” to this question they were then asked on how many days, and during the day how long, they walked or cycled for transport. Answers to these questions were translated into metabolic equivalents (METs), and the WHO recommendation of achieving at least 600 METs [18] used as the cut-off for individuals to be categorised as physically active.

Alcohol consumption is also self-reported, participants were asked if they consumed alcohol in the past 12 months, and then if so the frequency of consumption in the past week. For analyses individuals were classified as “non-drinkers” (had not consumed alcohol in the past 12 months, or did not report consuming alcohol in the previous week) or “drinkers” (reported consuming at least one alcoholic beverage in the past week).

Tobacco use was based on reported frequency of smoking tobacco (cigarettes) and/or using smokeless tobacco (for example snuff or chewing tobacco), in a similar manner to questions on physical activity and alcohol use. Individuals were also asked if they previously used tobacco. Therefore, this variable was categorised as “no reported tobacco use”, “past tobacco use” and “current tobacco use”.

### Analyses

Analyses for the population and dietary behaviour characteristics were performed on the sample of individuals with data on all three dietary behaviours from the seven countries. The complex survey design was accounted for, via the Stata *svy* command [39], and data were weighted so that data from each country contributed equally to the results. Percentages for categorical variables and means for continuous variables of demographic, behavioural and disease characteristics, by sex, were described and differences between sexes tested using Pearson’s chi-squared test for categorical variables and regression analysis for continuous variables.

Generalized linear models with country-level fixed effects were used to investigate cross-sectional associations between the dietary behaviours and waist circumference. Given that our outcome variables were discrete (i.e. dichotomous), we have fitted our generalized linear models using the binomial family distribution. For the hypertension and diabetes outcomes, separate multinomial logistic regression models with country-level fixed effects were used, comparing undiagnosed and self-reported diagnosed hypertension or diabetes with non-hypertensives or non-diabetics, respectively. For the waist circumference outcome models were adjusted for age, educational attainment, working status, physical activity, alcohol use and tobacco use. For the hypertension and diabetes outcomes, models were adjusted for age, educational attainment, working status, physical activity, alcohol use, tobacco use and waist circumference. Complete case analyses were conducted. Information on the number and proportion of participants with missing data on the outcome, independent or confounding variables is provided overall and by country in Additional file 1: Table S1.

To investigate the interaction of sex with the dietary behaviours on the outcomes, interaction terms were used and marginal estimates (proportion of males and females with the outcome for the dietary behaviour) were calculated. For these interactions a more lenient *p-* value of ≤0.10 was used to identify significance. Given the high proportion of respondents who reported using vegetable oil in cooking (93%) we have not presented the results by type of oil used, as findings were not informative. For the hypertension outcome two sensitivity analyses were conducted using the 4-point, and the 7-point salt behaviour score.

The results are presented with 95% confidence intervals. All analyses were conducted in Stata version 15.1 (StataCorp, College Station, Texas, US).

## Results

### Sample characteristics and dietary behaviours

The sample included 25,324 participants from Bhutan, Eswatini, Georgia, Guyana, Kenya, Nepal, and St Vincent and the Grenadines (Additional file 1: Table S2). The final analytic sample included 24,332 participants with the required information on the three dietary behaviours, of which 20,784 had waist circumference measurements, 22,907 had the required information on hypertension status, and 16,830 had the required information on diabetes status. Population characteristics are presented in Table 1, with characteristics for each outcome sample shown in Additional file 1: Table S3. Mean age was 36 years and 50% of the sample was female. On average, males were more likely to have had a formal education, to consume alcohol and to use tobacco (Table 1). For overall disease prevalence (95% CI), 26.0% (25.0–27.1%) of the sample analyzed had a high waist circumference, 11.0% (10.2–11.9%) of males and 41.4% (39.7–43.0%) of females. Just under a third of the sample were affected by hypertension (26.7%, 25.8–27.6% overall, 27.4%, 26.1–28.8% of males and 26.0%, 25.0–26.9% of females), 11.3% (10.8–11.8%) of which was self-reported diagnosed (8.7%, 8.1–9.4% of males, 13.8%, 13.1–14.5% of females) and 15.4% (14.7–16.2%) of which was undiagnosed (18.7%, 17.5–19.9% of males, 12.2%, 11.5–12.9% of females). Around 6 % of the sample had diabetes (5.8%, 5.2–6.5% overall, 4.9%, 4.3–5.7% of males, 6.7%, 5.9–7.5% of females), 3.4% (2.9–4.0%) reported being diagnosed with diabetes (2.6%, 2.1–3.2 of males, 4.1%, 3.5–4.9% of females) and 1.8% (1.5–2.1%) had undiagnosed diabetes (1.7%, 1.4–2.2% of males, 1.9%, 1.5–2.3% of females).

**Table 1:** Characteristics of individuals with data on dietary behaviours (*n* = 24,332) in seven low- and middle- income countries, overall and by sex ^a^

	Overall (95% CI)	Male (95% CI)	Female (95% CI)	*p*-value^*^
Socio-demographic characteristics				
Sex (%)				
Males	49.89 (48.81. 50.96)	–	–	
Females	50.11 (49.04, 51.18)	–	–	
Age (mean, years)	36.33 (36.03, 36.63)	36.24 (35.81, 36.66)	36.42 (36.08, 36.76)	0.47
Educational Attainment (%)				
No formal schooling	14.79 (13.48, 16.20)	11.29 (9.96, 12.77)	18.26 (16.64, 20.01)	< 0.001
Primary school	30.51 (29.18, 31.88)	32.25 (30.42, 34.14)	28.78 (27.53, 30.07)	
Secondary school or above	54.70 (53.23, 56.17)	56.46 (54.45, 58.45)	52.95 (51.37, 54.53)	
Working (%)	54.18 (52.50, 55.83)	68.74 (66.98, 70.45)	39.70 (37.29, 42.16)	< 0.001
Behavioural characteristics				
Physical Activity (%)				
Achieving 600 MET a week	84.50 (82.61, 86.21)	88.92 (87.62, 90.11)	80.10 (77.30, 82.63)	< 0.001
Alcohol consumption				
Mean number of drinks per week	3.84 (3.45, 4.24)	6.47 (5.80, 7.15)	1.23 (1.00, 1.45)	< 0.001
Consuming alcohol during a week (%)				
No alcohol use reported	70.65 (69.26, 71.99)	56.15 (54.29, 57.99)	85.06 (83.62, 86.40)	< 0.001
Consume one alcoholic drink or more	29.35 (28.01, 30.74)	43.85 (42.01, 45.71)	14.94 (13.60, 16.38)	
Tobacco use, smoke or smokeless (%)				
No tobacco use	69.69 (68.31, 71.04)	51.51 (49.54, 53.49)	87.79 (86.84, 88.68)	< 0.001
Past use of tobacco	19.29 (18.13, 20.50)	32.15 (30.300, 34.05)	6.48 (5.83, 7.19)	
Current use of tobacco	11.02 (10.34, 11.74)	16.33 (15.21, 17.53)	5.73 (5.18, 6.33)	
Cardiovascular risk factors				
Waist circumference				
Mean waist circumference	85.22 (84.76, 85.68)	84.45 (83.98, 84.92)	86.01 (85.34, 86.68)	< 0.001
High waist circumference (%) ^b^	26.01 (24.96, 27.08)	11.02 (10.20, 11.89)	41.35 (39.73, 43.00)	< 0.001
Blood pressure measures				
Mean systolic blood pressure	125.83 (125.47, 126.19)	128.47 (127.96, 128.97)	123.21 (122.76, 123.67)	< 0.001
Mean diastolic blood pressure	79.76 (79.39, 80.13)	79.90 (79.39, 80.41)	79.62 (79.26, 79.98)	0.26
Hypertension (%) ^c^	26.69 (25.82, 27.58)	27.44 (26.12, 28.81)	25.95 (25.01, 26.92)	0.05
Self-reported diagnosed hypertension	11.26 (10.76, 11.79)	8.74 (8.10, 9.43)	13.77 (13.06, 14.51)	< 0.001
Undiagnosed hypertension	15.43 (14.71, 16.18)	18.70 (17.54, 19.92)	12.18 (11.49. 12.91)	
Blood glucose measures				
Mean blood glucose measure	4.83 (4.79, 4.87)	4.79 (4.74, 4.83)	4.87 (4.82, 4.93)	0.006
Diabetes (%) ^d^	5.82 (5.23, 6.47)	4.94 (4.30, 5.66)	6.66 (5.92, 7.49)	< 0.001
Self-reported diagnosed diabetes	3.38 (2.86, 3.99)	2.59 (2.10, 3.19)	4.13 (3.45, 4.93)	< 0.001
Undiagnosed diabetes	1.79 (1.53, 2.10)	1.72 (1.35, 2.18)	1.86 (1.54, 2.25)	

^a^ Percentages and means accounts for sampling design with survey weights re-scaled by the survey’s sample size such that all countries contribute equally to estimates. Differences between sexes tested using Pearson’s chi-squared test for categorical variables and linear regression analysis for continuous variables

^b^Definition of high waist circumference, waist ≥102 cm for males and waist ≥88 cm for females

^c^ Hypertension was defined as an average systolic blood pressure (SBP) measurement > 140 mmHg, or their average diastolic blood pressure (DBP) measurement > 90 mmHg, or they reported taking medication for hypertension. Self-reported diagnosed hypertension were those who met the criteria for hypertension and also reported a diagnosis of hypertension. Undiagnosed individuals were those who had a high SBP (> 140 mmHg) or a high DBP (> 90 mmHg), did not report taking hypertension medication, and did not report a hypertension diagnosis

^d^ Diabetes was defined as having an average fasting blood glucose (FBG) level ≥ 7 mmol/L, or having a random blood glucose (RBG) level of ≥11.1 mmol/L or on medication for diabetes. Individuals with self-reported diagnosed diabetes met the criteria for diabetes and also reported a diagnosis of diabetes. Undiagnosed individuals were those who had a high FBG (≥7 mmol/L) or a high RBG (≥11.1 mmol/L), did not report taking diabetes medication, and did not report a diabetes diagnosis

^*^p-value for difference between males and females

**Fig. 1:**
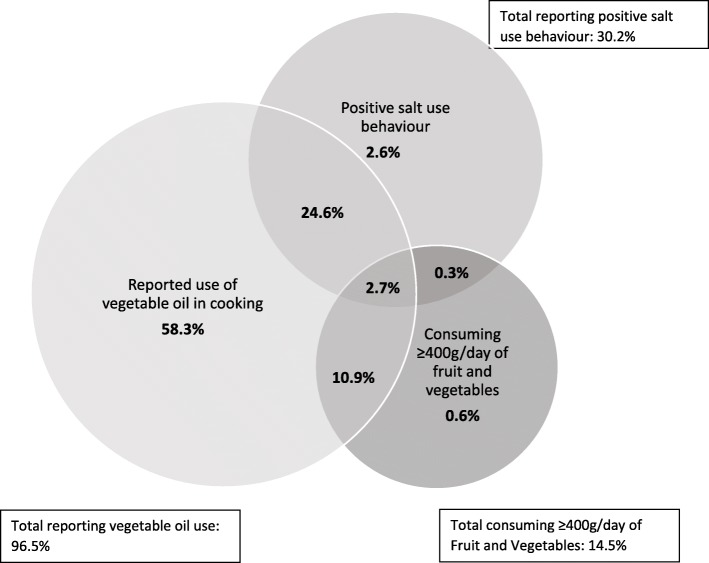
Weighted proportion of participants reporting positive dietary behaviours (*n* = 23,511), in seven low-and middle-income countries

**Fig. 2:**
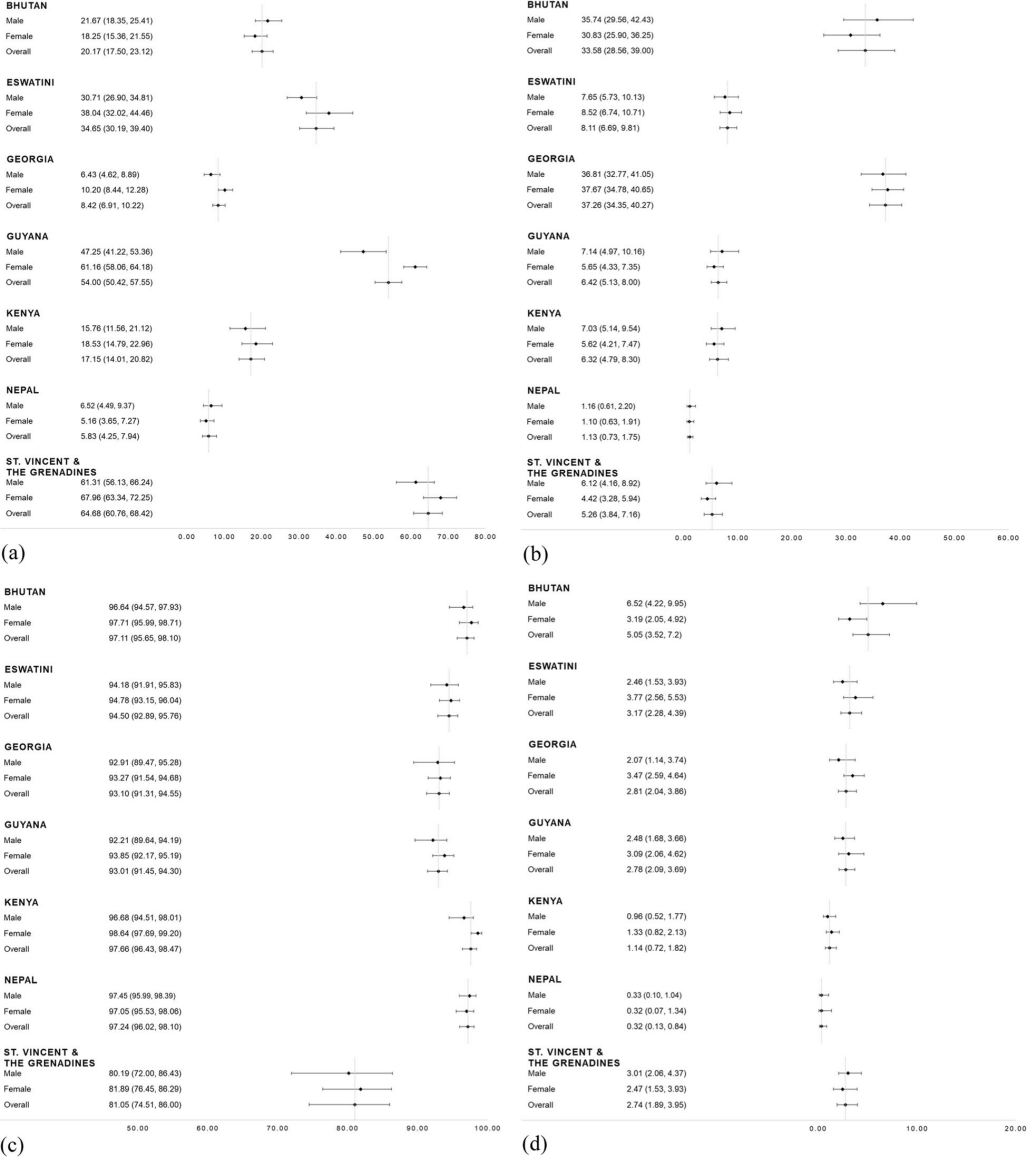
Prevalence (percentage, 95% confidence interval) of (**a**) reporting positive salt use behaviour, (**b**) meeting fruit and vegetable recommendations, (**c**) use of vegetable oil, and (**d**) reporting all three behaviours positively, by sex and country

A third of the sample (29.3, 95% CI 26.8–31.9%) reported positive salt use behaviour, slightly higher in females than in males (31.3%, 28.6–34.2% compared to 27.2%, 24.6–30.0%, *p*-value< 0.001 Table 2). Analysis of the salt behaviour from the seven questions asked in the survey revealed a higher proportion of participants responded positively to questions regarding adding salt to meals (*never*, 53.1%, 50.9–55.3%) and limiting processed foods to reduce salt intake (*yes*, 43.3%, 40.9–45.7%). However, 63.8% (61.9–65.7%) of the population reported always adding salt during cooking and 18.0% (16.7–19.4%) reported looking at the salt content on food labels. Fourteen percent (14.0%, 12.8–15.3%) of the sample met the WHO fruit and vegetable recommendations, with a lower proportion of females meeting the recommendations compared to males (13.2%, 12.1–14.4% compared to 14.8%, 13.2–16.6%, *p*-value = 0.02). The majority of the sample reported using vegetable oil in cooking (93.4%, 92.2–94.4%, Table 2). Overall, 2.7% of the population reported positive behaviours for all three dietary factors (Fig. 1), with no sex differences evident (Additional file 1: Figure S1).The prevalence of positive dietary behaviours was similar for each outcome population (Additional file 1: Table S4). The prevalence of positive dietary behaviours varied by country (Fig. 2), ranging from 64.7% (60.8–68.4%) reporting positive salt behaviour in St. Vincent & the Grenadines to 5.8% (4.3–7.9%) reporting positive salt use behaviour in Nepal (Fig. 2a), and 37.3% (34.4–40.3%) reporting meeting fruit and vegetable recommendations in Georgia to 1.1% (0.7–1.8%) meeting fruit and vegetable recommendations in Nepal (Fig. 2b).

**Table 2:** Self-reported salt use behaviour, fruit and vegetable consumption and the type of fat and oil used in cooking, in seven low-and middle-income countries (*n* = 24,332), by sex ^a^

	Overall Percentage (95% CI)	Male Percentage (95% CI)	Female Percentage (95% CI)	*p*-value^*^
Salt use behaviour				
Positive salt behaviour (> 50%)	29.27 (26.75, 31.93)	27.19 (24.60, 29.95)	31.34 (28.61, 34.21)	< 0.001
Specific salt behaviours				
Add salt to meal				< 0.001
Always	8.44 (7.64, 9.32)	9.39 (8.26, 10.66)	7.50 (6.74, 8.33)	
Often	5.28 (4.76, 5.85)	5.27 (4.62, 6.01)	5.28 (4.69, 5.95)	
Sometimes	17.35 (16.45, 18.28)	18.09 (16.86, 19.39)	16.61 (15.60, 17.67)	
Rarely	15.83 (14.58, 17.16)	16.17 (14.64, 17.82)	15.49 (14.16, 16.93)	
Never	53.10 (50.92, 55.27)	51.08 (48.69, 53.47)	55.11 (52.76, 57.44)	
Add salt during cooking				0.26
Always	63.78 (61.88, 65.65)	63.48 (61.28, 65.62)	64.09 (62.07, 66.06)	
Often	7.62 (6.95, 8.35)	7.58 (6.72, 8.53)	7.67 (6.96, 8.45)	
Sometimes	11.34 (10.50, 12.24)	11.74 (10.65, 12.92)	10.95 (10.05, 11.92)	
Rarely	7.59 (6.91, 8.32)	7.21 (6.35, 8.18)	7.95 (7.20, 8.78)	
Never	9.67 (8.75, 10.67)	10.00 (8.84, 11.29)	9.34 (8.39, 10.38)	
Limit Processed foods to reduce salt				
Yes	43.3 (40.94, 45.70)	42.35 (39.78, 44.96)	44.25 (41.67, 46.87)	0.07
Look at salt content on food labels				
Yes	18.03 (16.71, 19.42)	16.96 (15.51, 18.51)	19.09 (17.56, 20.72)	0.01
Buy low salt alternatives				
Yes	18.16 (16.69, 19.72)	16.81 (15.17, 18.59)	19.49 (17.89, 21.21)	< 0.001
Use other spices				
Yes	32.94 (29.72, 36.34)	31.24 (28.16, 34.48)	34.64 (31.00, 38.64)	< 0.001
Avoid eating foods prepared outside of home				
Yes	34.05 (31.92, 36.24)	31.34 (29.12, 33.65)	36.74 (34.36, 39.19)	< 0.001
Fruit and vegetable consumption				
Met WHO guidelines (400 g per day)	14.01 (12.80, 15.32)	14.81 (13.23, 16.55)	13.21 (12.09, 14.43)	0.02
Fat and oil used in cooking				0.45
Vegetable	93.39 (92.20, 94.40)	92.95 (91.48, 94.19)	93.81 (92.69, 94.77)	
Animal	2.49 (2.02, 2.06)	2.62 (2.00, 3.41)	2.36 (1.92, 2.89)	
Other	2.98 (2.14, 4.14)	3.17 (2.15, 4.65)	2.78 (2.04, 3.78)	
None in particular	0.47 (0.36, 0.61)	0.49 (0.33, 0.71)	0.45 (0.33, 0.62)	
None	0.69 (0.51, 0.91)	0.77 (0.51, 0.12)	0.60 (0.45, 0.80)	

^*^p-value for difference between males and females

^a^ Percent accounts for sampling design with survey weights re-scaled by the survey’s sample size such that all countries contribute equally to estimates. Differences between sexes tested using Pearson’s chi-squared test

Individuals with missing data for the diabetes outcome were compared to individuals with data in an unweighted analysis. Those with data were older (39 vs. 36 years), had a higher mean waist circumference (88.28 vs. 85.11 cm), had a higher average systolic (129.78 vs. 125.19 mmHg) and diastolic blood pressure (81.68 vs. 79.21 mmHg), a higher proportion were hypertensive (17.6 vs. 12.6%), and had higher average blood glucose levels (5.73 vs. 4.27 mmol/L). However, no differences were evident in the reported dietary behaviours. The proportion of participants with missing data from the hypertension and waist circumference outcome groups were minimal, 321 (1.4%) and 1059 (4.4%) participants, respectively Additional file 1: Table S3.

### Cross-sectional associations of sex and dietary behaviours with waist circumference, hypertension, and diabetes

From the adjusted models (adjusted for age, waist circumference (for associations with diabetes and hypertension), educational attainment, working status, physical activity, alcohol use and tobacco use) a higher proportion of females exceeded waist circumference recommendations in comparison to males (40.5, 95% CI 35.6–45.4% vs. 10.1, 6.6–13.5%). For hypertension, a higher proportion of males had undiagnosed hypertension in comparison to females (19.2%, 17.8–20.7% vs. 12.2%, 11.0–13.5%), with no difference in the proportion with diagnosed hypertension between the sexes (10.7%, 9.8–11.6% for males, 11.7%, 10.9–12.4% for females). For diabetes, there were no sex differences in the proportion with undiagnosed or diagnosed diabetes (undiagnosed diabetes, 2.1%, 1.6–2.6% of males, 1.7%, 1.4–2.0% of females, diagnosed diabetes, 8.3%, 7.4–9.2% of males, 7.0, 6.7–7.4% of females).

Overall, salt behaviour was associated with diagnosed diabetes only (Table 3). A higher proportion of those with diagnosed diabetes reported positive salt use behaviour, compared to those who reported poor salt behaviour (8.0, 95% CI 7.9–8.2% vs. 6.5%, 6.3–6.8% respectively, *p*-value = 0.001). However, when looking at the interaction by sex there were further significant differences (Table 3). For undiagnosed hypertension there was a significant interaction by sex (*p*-value for interaction = 0.04), the proportion of females with undiagnosed hypertension reporting poor salt behaviour was 13.1% (11.8–14.4%) compared to 9.9% (8.4–11.5%) of those who reported positive salt behaviour. However, in males there was no difference in the proportion of undiagnosed hypertension for those who reported positive or poor salt behaviour. Salt behaviour was also associated with undiagnosed diabetes, with a significant interaction by sex (*p*-value for interaction = 0.02). The proportion of males with undiagnosed diabetes reporting poor salt behaviour was 2.4% (2.0–2.9%) compared to 1.5% (0.6–2.4%) for those who reported positive salt behaviour, yet there was no difference in the prevalence of undiagnosed diabetes by salt behaviour for females. In the sensitivity analyses (Additional file 1: Figure S2 and S3) a downwards trend was seen for the prevalence of undiagnosed hypertension with increasing numbers of salt behaviour questions answered positively for females. Comparatively, for males a slight upward trend was seen for both the 7-point and the 4-point scores. In both cases, the confidence intervals for each prevalence-point overlapped.

**Table 3:** Cross-sectional associations of salt behaviour with exceeding waist circumference ^a^ recommendations, having undiagnosed or diagnosed hypertension ^b^ or diabetes ^b^, in seven low-and middle-income countries

	Waist circumference ^c^ (*n* = 20,784)	Hypertension ^d^ (*n* = 22,907)		Diabetes ^e^ (*n* = 16,643)	
	Percentage (95% CI) exceeding recommendations	Percentage (95% CI) undiagnosed	Percentage (95% CI) diagnosed	Percentage (95% CI) undiagnosed	Percentage (95% CI) diagnosed
Overall					
good salt behaviour	24.3 (22.3, 26.2)	14.8 (13.1, 16.4)	12.1 (11.1, 13.1)	1.7 (1.3, 2.2)	8.0 (7.9, 8.2)
poor salt behaviour	22.3 (21.4, 23.1)	16.0 (15.5, 16.6)	10.9 (10.5, 11.4)	1.9 (1.7, 2.2)	6.5 (6.3, 6.8)
*p-value*	*0.81*	*0.67*	*0.34*	*0.20*	*0.001*
Male					
good salt behaviour	10.4 (7.7, 13.1)	19.7 (15.2, 24.1)	11.5 (9.9, 13.0)	1.5 (0.6, 2.4)	9.3 (8.5, 10.2)
poor salt behaviour	9.9 (5.4, 14.3)	19.1 (18.4, 19.8)	10.3 (9.0, 11.7)	2.4 (2.0, 2.9)	6.8 (5.4, 8.1)
Female					
good salt behaviour	43.3 (38.3, 48.4)	9.9 (8.4, 11.5)	12.5 (11.8, 13.2)	1.9 (1.6, 2.2)	7.3 (6.9, 7.7)
poor salt behaviour	39.3 (33.9, 44.7)	13.1 (11.8, 14.4)	11.3 (10.2, 12.4)	1.6 (1.2, 2.0)	6.4 (5.4, 7.4)
*p-value for sex interaction*	*0.64*	*0.04*	*0.79*	*0.02*	*0.29*

^a^ Model adjusted for type of fat and oil used in cooking, age, education, working status, physical activity, alcohol use and tobacco use

^b^ Model adjusted for type of fat and oil used in cooking, age, education, working status, physical activity, alcohol use, tobacco use and waist circumference

^c^ Definition of high waist circumference, waist ≥102 cm for males and waist ≥88 cm for females

^d^ Hypertension was defined as an average systolic blood pressure (SBP) measurement > 140 mmHg, or their average diastolic blood pressure (DBP) measurement > 90 mmHg, or they reported taking medication for hypertension. Self-reported diagnosed hypertension were those who met the criteria for hypertension and also reported a diagnosis of hypertension. Undiagnosed individuals were those who had a high SBP (> 140 mmHg) or a high DBP (> 90 mmHg), did not report taking hypertension medication, and did not report a hypertension diagnosis

^e^ Diabetes was defined as having an average fasting blood glucose (FBG) level ≥ 7 mmol/L, or having a random blood glucose (RBG) level of ≥11.1 mmol/L or on medication for diabetes. Individuals with self-reported diagnosed diabetes met the criteria for diabetes and also reported a diagnosis of diabetes. Undiagnosed individuals were those who had a high FBG (≥7 mmol/L) or a high RBG (≥11.1 mmol/L), did not report taking diabetes medication, and did not report a diabetes diagnosis

Overall, self-reported fruit and vegetable consumption was associated with waist circumference (Table 4), with a higher proportion of those who met fruit and vegetable recommendations exceeding waist circumference recommendations (24.4, 95% CI 22.5–26.4% vs 22.6%, 22.3–23.0% respectively, *p*- value = 0.01). At the *p-*value *≤*0.10 significance level a significant interaction was observed by sex for fruit and vegetable consumption with waist circumference (*p*-value for interaction = 0.06), with a higher proportion of males who met fruit and vegetable recommendations exceeding waist circumference recommendations (13.1%, 6.6–19.6% compared to 9.5%, 6.6–12.4%). There was no difference in the prevalence of high waist circumference by fruit and vegetable consumption for females. No associations were identified between fruit and vegetable consumption and prevalence of undiagnosed or diagnosed hypertension (*p*-values of 0.84 and 0.88, respectively), or the prevalence of undiagnosed or diagnosed diabetes (*p*-values 0.75 and 0.33, respectively). Further, no significant interactions by sex were found (*p*-values 0.17 for undiagnosed hypertension, 0.79 for diagnosed hypertension, 0.97 for undiagnosed diabetes and 0.90 for diagnosed diabetes).

**Table 4:** Cross-sectional associations of meeting fruit and vegetable recommendations with exceeding waist circumference recommendations ^a^, having undiagnosed or diagnosed hypertension ^b^ or diabetes ^b^, in seven low-and middle-income countries

	Waist circumference ^c^ (*n* = 20,784)	Hypertension ^d^ (*n* = 22,907)		Diabetes ^e^ (*n* = 16,643)	
	Percentage (95% CI) exceeding recommendations	Percentage (95% CI) undiagnosed	Percentage (95% CI) diagnosed	Percentage (95% CI) undiagnosed	Percentage (95% CI) diagnosed
Overall					
Met F&V^f^ recommendations	24.4 (22.5, 26.4)	15.9 (13.8, 18.0)	11.2 (10.2, 12.2)	1.8 (0.7, 2.9)	5.9 (4.5, 7.2)
Did not meet F&V recommendations	22.6 (22.3, 23.0)	15.6 (15.3, 16.0)	11.3 (11.1, 11.5)	1.9 (1.8, 2.0)	7.5 (7.4, 7.6)
*p-value*	*0.01*	*0.84*	*0.88*	*0.75*	*0.33*
Male					
Met F&V recommendations	13.1 (6.6, 19.6)	18.9 (17.0, 20.9)	10.6 (9.4, 11.7)	2.0 (1.0, 3.0)	6.5 (3.1, 9.9)
Did not meet F&V recommendations	9.5 (6.6, 12.4)	19.3 (17.4, 21.1)	10.7 (9.5, 12.0)	2.1 (1.5, 2.7)	8.4 (7.5, 9.4)
Female					
Met F&V recommendations	39.8 (32.5, 47.1)	13.1 (10.7, 15.4)	11.6 (10.5, 12.8)	1.6 (0.2, 3.3)	5.6 (4.2, 6.9)
Did not meet F&V recommendations	40.6 (35.9, 45.3)	12.1 (11.0, 13.2)	11.7 (11.0, 12.4)	1.7 (1.5, 2.0)	7.1 (6.5, 7.6)
*p-value for sex interaction*	*0.06*	*0.17*	*0.79*	*0.97*	*0.90*

^a^Model adjusted for type of fat and oil used in cooking, age, education, working status, physical activity, alcohol use and tobacco use

^b^Model adjusted for type of fat and oil used in cooking, age, education, working status, physical activity, alcohol use, tobacco use and waist circumference

^c^ Definition of high waist circumference, waist ≥102 cm for males and waist ≥88 cm for females

^d^ Hypertension was defined as an average systolic blood pressure (SBP) measurement > 140 mmHg, or their average diastolic blood pressure (DBP) measurement > 90 mmHg, or they reported taking medication for hypertension. Self-reported diagnosed hypertension were those who met the criteria for hypertension and also reported a diagnosis of hypertension. Undiagnosed individuals were those who had a high SBP (> 140 mmHg) or a high DBP (> 90 mmHg), did not report taking hypertension medication, and did not report a hypertension diagnosis

^e^ Diabetes was defined as having an average fasting blood glucose (FBG) level ≥ 7 mmol/L, or having a random blood glucose (RBG) level of ≥11.1 mmol/L or on medication for diabetes. Individuals with self-reported diagnosed diabetes met the criteria for diabetes and also reported a diagnosis of diabetes. Undiagnosed individuals were those who had a high FBG (≥7 mmol/L) or a high RBG (≥11.1 mmol/L), did not report taking diabetes medication, and did not report a diabetes diagnosis

^f^ “F&V” – Fruit and vegetable intake, categorised into meeting or not meeting fruit and vegetable recommendations of 400 g/day

## Discussion

This study revealed an exceptionally low prevalence of positive dietary behaviours for salt use and fruit and vegetable consumption, with only 2.7% of the population reporting positive salt use, meeting fruit and vegetable recommendations and reporting use of vegetable oil in cooking. Small sex differences were evident in the self-report of salt use and fruit and vegetable consumption, but associations between the self-reported dietary behaviours and the outcomes were minimal. This was unexpected but can likely be explained by the low prevalence of positive dietary behaviours overall.

The results for positive salt use behaviour and meeting the WHO recommendations for fruit and vegetables varied hugely by country. 64.7% of the population from St Vincent & the Grenadines reported positive salt use behaviour, and 37.3% of the Georgian population met fruit and vegetable recommendations, compared to just 5.8 and 1.1% of the Nepalese population for the respective behaviours. Across the countries, discretionary salt use was high, with 63% of the sample *always adding salt during cooking*. These responses suggest discretionary salt is a key contributor to salt intake in these countries [40–42]. We found a small proportion of participants reported looking at the salt content on food labels (18% overall, 17% of males and 19% of females). This is much lower than that found in two separate reviews of nutrition label use in other low-and-middle income countries [43] and in high-income countries [44], finding 40–70% and 60–80% self-reported use, respectively. Both of these reviews found that self-reported use of labels was high, comprehension of back-of-pack nutrition panels was low, and interpretative front-of-pack labels, for example the multiple traffic light label, were easier to understand, making it more likely to influence consumer choice. As consumption of processed foods increases, it is important that clear and effective labelling systems are introduced. Monitoring of the main sources of salt in diets is also needed [45], to inform future intervention strategies. The identified low fruit and vegetable consumption across the countries, echoes findings by Frank et al. [31] and the Prospective Urban and Rural Epidemiological (PURE) Study [14, 46]. However, the PURE study [46], which covers 18 counties did identify a decrease in cardiovascular disease with increasing fruit, vegetable and legume intake. Differing LMICs included in studies, the lack of legume measurement in WHO STEPS and the cross-sectional nature of studies in our review potentially explain the differing findings.

Our findings imply poor overall diet quality in the included countries, particularly for Nepal, Kenya and Eswatini, where the prevalence of meeting fruit and vegetable recommendations and reporting positive salt use behaviors were very low. The recent review on the State of Diet Quality Globally [47] looked at unhealthy and healthy dietary patterns using the 2015 Global Dietary Database. The authors found that adherence to both “unhealthy” and “healthy” dietary patterns were low in Nepal, Kenya and Eswatini. Their unhealthy dietary pattern score was based on the consumption of refined grains, total processed meats, sugar-sweetened beverages and added sugar, where as their healthy dietary pattern score focused on 11 dietary factors including fruits, vegetables, legumes, wholegrains and unprocessed animal products. These results further highlight the need to increase “healthy” foods, including fruits and vegetables. Accessibility, affordability, and safety of fruits and vegetables are key barriers to consumption in low-resource settings [14, 48], and policies that focus on contextually appropriate systems, fostering production of fruits and vegetables by local farmers, and proper storage and handling of produce to point of sale, at potentially subsidized prices may aid consumption [49, 50].

Examination of cross-sectional associations of the dietary behaviours with outcomes produced differing results for males and females. For waist circumference, once adjusted for socioeconomic and behavioural factors, 41% of females exceeded waist circumference recommendations, compared to 10% of males. Our findings are consistent with the obesity transition where females tend to transition to obesity before males [4, 6]. Individuals who met fruit and vegetable recommendations were more likely to exceed waist circumference recommendations. Whilst we were not able to adjust for total energy intake, it is highly likely that this is because people who meet fruit and vegetable recommendations may eat more in general. It is also acknowledged that the use of waist circumference cut-offs have their limitations, and different cut-offs exist for different populations [51, 52]. We have used binary variables in this paper for ease of interpretation, however cut-offs, either for waist circumference or the categories of body mass index may not predict the same disease risk for all population groups. Therefore, we could be overestimating the burden of high-waist circumference in our sample, which is inclusive of a range of ethnicities. We found that poor self-reported salt behaviour was associated with increased odds of having undiagnosed hypertension for females, with no relationship evident for males. This is interesting as some sodium reduction trials also show that reducing sodium has more of an impact on blood pressure in females than males [53]. Given we cannot equate the behavioural questionnaire in the present study to actual sodium intake, a next step investigation could be to examine the association of the salt behaviour questions included in STEPS surveys with actual salt intake measured by 24-h urine/spot urine, which has been measured in recent STEPS surveys. The fact that a higher proportion of males with poor salt behaviour had undiagnosed diabetes compared to males with good salt behaviour was intriguing, albeit the percentage difference between the groups was only 0.9%. The relationship between salt intake and diabetes is not well established, however it is likely to be associated given diets high in salt may also be energy dense, leading to excess adiposity and therefore risk of type 2 diabetes [54, 55].

Overall, it is important to reflect on the dietary behaviours measured in the STEPS survey given that for many LMICs, the STEPS surveys are the only source of national dietary intake data. In particular, ultra-processed foods and drinks are important overlooked dietary risk factors [56, 57] and countries should consider including questions on these in future iterations of the STEPS survey. These products are high in salt, fat and/or sugar, and people who frequently consume ultra-processed products in their diets often have low intakes of fresh fruits and vegetables [57]. Sales of ultra-processed products have been shown to be increasing globally, including in LMICs, with corresponding increases in body mass index [58]. While we have investigated components of diet quality, we were not able to investigate the level of consumption of ultra-processed products, which may be a reason for the overall minimal associations observed between the diet behaviours and cardiovascular risk factors.

These findings have several policy implications for the included countries. First, they identify the need to improve consumption of fruits and vegetables, and salt use behaviour. As discussed, policies need to focus on improving the accessibility and affordability of fruit and vegetables, and decreasing the use of salt during cooking, while monitoring the consumption of ultra-processed products which are becoming more accessible in LMICs. Second, there is not sufficient evidence from this review to support the idea that we need sex specific policies and interventions for fruit and vegetable consumption and salt use. This investigation was limited by the small proportion of individuals reporting positive fruit and vegetable consumption and salt use behaviour. If future policies are implemented to improve dietary behaviours it would be worthwhile investigating effectiveness by sex, in addition to overall effectiveness. Given that WHO STEPS surveys are regularly conducted, they can be used to monitor policy effectiveness and a similar study to the present could be conducted as a method of monitoring and evaluation in individual countries.

The strengths of our study are that to our knowledge, this is the first study that has examined sex differences in dietary behaviours and their association with CVD risk factors in multiple LMICs. The study pooled data from 7 nationally representative surveys, across 7 countries meaning 24,332 people were included in the analysis. Given all of these surveys were STEPS surveys they used the same standardised methodology to measure all variables included in the present analyses. Additionally, in country collaborators are authors on the present study, and therefore were able to aid interpretation of our results by adding contextual information in addition to their oversight of the development of this paper. However, our study has several weaknesses. First, the data is cross-sectional and therefore the associations discussed do not imply causation. Second, only seven STEPS surveys were included as only more recent STEPS surveys have included dietary behaviour questions. It would be worthwhile to rerun this analysis in coming years as more countries collect this data. Third, 93.4% of the study sample reported the use of vegetable oil and therefore it was not useful to include an analysis of the cross-sectional association of oil type used with CVD risk factors in our results. This question has since been removed by WHO in the updated version of the STEPS survey questionnaire [21], on this basis. Finally, the dietary behaviour questions analysed do not provide a comprehensive picture of an individual’s diet, and do not allow for the quantification of dietary intake. Additionally, the self-report of dietary behaviours is subject to multiple biases [59]. While overall dietary intake is not assessed by STEPS surveys, the survey has been used widely throughout low-and middle-income countries to assess risk of non-communicable disease based on the key dietary behaviours. This provides useful insight on the need for dietary interventions at a population level in resource poor settings [34]. Urinary markers of sodium intake have been collected in more recent STEPS surveys [21, 45], however these data were not available for the current project.

## Conclusion

In conclusion, just 2.7% of respondents from seven countries in this study reported positive behaviours for salt use, fruit and vegetable consumption and use of vegetable oil in cooking, with variability seen by country. Given the high burden of cardiovascular diseases in the countries studied, there is an urgent need to implement suitable policies to encourage greater intake of fruit and vegetables and reduced consumption of salt. We identified small sex differences in the self-report of salt use behaviour and fruit and vegetable consumption, along with some interesting interactions by sex with the dietary behaviours for having a high waist circumference, hypertension or diabetes. As such our evidence is not sufficient to endorse the tailoring of diet related interventions by sex in the included countries as our findings were limited by the small proportion of the population reporting positive dietary behaviours. However, if adherence to healthy diets were greater it is plausible that greater associations and sex differences would have been identified, and therefore this hypothesis should be a focus of future research.

## Supplementary information

**Additional file 1: Table S1.** Number and percent of individuals with missing data on outcome, independent variables and confounders*, overall and by country. **Table S2.** Survey characteristics. **Table S3.** Characteristics of individuals with data on dietary behaviours, and data on waist circumference (*n* = 23,273) hypertension (*n* = 24,011), diabetes (*n* = 17,724) status. **Table S4.** Prevalence of dietary behaviours among participants with data on waist circumference (*n* = 23,273) hypertension (*n* = 24,011), or diabetes (*n* = 17,724) status. **Figure S1**. a**.** Weighted proportion of males (*n* = 8551) reporting positive dietary behaviours, in seven low-and middle-income countries. b**.** Weighted proportion of females (*n* = 14,960) reporting positive dietary behaviours, in seven low-and middle-income countries. **Figure S2.** a. Percentage (95% confidence interval) of males with undiagnosed hypertension, by the self-report of salt behaviour on a seven point scale*. b**.** Percentage (95% confidence interval) of females with undiagnosed hypertension, by the self-report of salt behaviour on a seven point scale*. **Figure S3.** a. Percentage (95% confidence interval) of males with undiagnosed hypertension, by the self-report of salt behaviour on a four point scale*.b**.** Percentage (95% confidence interval) of females with undiagnosed hypertension, by the self-report of salt behaviour on a four point scale*.

## Data Availability

The datasets used and/or analysed during the current study are available from the corresponding author on reasonable request.
