# Supplementary material for: Evaluation of sex differences in dietary behaviours and their relationship with cardiovascular risk factors: a cross-sectional study of nationally representative surveys in seven low- and middle-income countries
Source: Nutr J. 2020 Jan 13;19:3. doi: 10.1186/s12937-019-0517-4 (PMC6956488; doi:10.1186/s12937-019-0517-4)
Supplement: Supplementary file 1 — Additional file 1: Table S1. Number and percent of individuals with missing data on outcome, independent variables and confounders*, overall and by country. Table S2. Survey characteristics. Table S3. Characteristics of individuals with data on dietary behaviours, and data on waist circumference (n = 23,273) hypertension (n = 24,011), diabetes (n = 17,724) status. Table S4. Prevalence of dietary behaviours among participants with data on waist circumference (n = 23,273) hypertension (n = 24,011), or diabetes (n = 17,724) status. Figure S1. a. Weighted proportion of males (n = 8551) reporting positive dietary behaviours, in seven low-and middle-income countries. b. Weighted proportion of females (n = 14,960) reporting positive dietary behaviours, in seven low-and middle-income countries. Figure S2. a. Percentage (95% confidence interval) of males with undiagnosed hypertension, by the self-report of salt behaviour on a seven point scale*. b. Percentage (95% confidence interval) of females with undiagnosed hypertension, by the self-report of salt behaviour on a seven point scale*. Figure S3. a. Percentage (95% confidence interval) of males with undiagnosed hypertension, by the self-report of salt behaviour on a four point scale*.b. Percentage (95% confidence interval) of females with undiagnosed hypertension, by the self-report of salt behaviour on a four point scale*. [file 12937_2019_517_MOESM1_ESM.docx]

Table S1. Number and percent of individuals with missing data on outcome, independent and confounding variables*, overall and by country

|  | Overall | | Bhutan | | Eswatini | | Georgia | | Guyana | | Kenya | | Nepal | | St. Vincent & the Grenadines | |
| --- | --- | --- | --- | --- | --- | --- | --- | --- | --- | --- | --- | --- | --- | --- | --- | --- |
|  | n | % | n | % | n | % | n | % | n | % | n | % | n | % | n | % |
| Waist circumference | 1,367 | 5.40 | 79 | 0.31 | 447 | 1.77 | 296 | 1.17 | 115 | 0.45 | 321 | 1.27 | 60 | 0.24 | 49 | 0.19 |
| Hypertension | 607 | 2.40 | 6 | 0.02 | 340 | 1.34 | 160 | 0.63 | 13 | 0.05 | 60 | 0.24 | 17 | 0.07 | 11 | 0.04 |
| Diabetes | 7,047 | 27.83 | 86 | 0.34 | 652 | 2.57 | 1,231 | 4.86 | 1,993 | 7.87 | 381 | 1.50 | 370 | 1.46 | 2,334 | 9.22 |
| Salt use behaviour | 261 | 1.03 | 3 | 0.01 | 255 | 1.01 | 0 | 0.00 | 0 | 0.00 | 3 | 0.01 | 0 | 0.00 | 0 | 0.00 |
| Fruit and vegetable consumption | 933 | 3.68 | 40 | 0.16 | 380 | 1.50 | 23 | 0.09 | 45 | 0.18 | 270 | 1.07 | 94 | 0.37 | 81 | 0.32 |
| Type of oil used in cooking | 326 | 1.29 | 3 | 0.01 | 263 | 1.04 | 35 | 0.14 | 4 | 0.02 | 9 | 0.04 | 0 | 0.00 | 12 | 0.05 |
| Sex | 0 | 0.00 | 0 | 0.00 | 0 | 0.00 | 0 | 0.00 | 0 | 0.00 | 0 | 0.00 | 0 | 0.00 | 0 | 0.00 |
| Age | 21 | 0.08 | 3 | 0.01 | 0 | 0.00 | 0 | 0.00 | 0 | 0.00 | 16 | 0.06 | 0 | 0.00 | 2 | 0.01 |
| Educational attainment | 496 | 1.96 | 3 | 0.01 | 256 | 1.01 | 229 | 0.90 | 2 | 0.01 | 0 | 0.00 | 0 | 0.00 | 6 | 0.02 |
| Working status | 268 | 1.06 | 2 | 0.01 | 257 | 1.01 | 6 | 0.02 | 0 | 0.00 | 0 | 0.00 | 0 | 0.00 | 3 | 0.01 |
| Physical activity | 289 | 1.14 | 3 | 0.01 | 259 | 1.02 | 0 | 0.00 | 1 | 0.00 | 4 | 0.02 | 22 | 0.09 | 0 | 0.00 |
| Alcohol consumption | 318 | 1.26 | 19 | 0.08 | 269 | 1.06 | 14 | 0.06 | 1 | 0.00 | 8 | 0.03 | 0 | 0.00 | 7 | 0.03 |

*Percent missing data on outcomes (waist circumference, hypertension and diabetes), independent variables (self-reported salt use behaviour, fruit and vegetable consumption and type of oil used in cooking) and potential confounding factors (age, educational attainment, working status, physical activity and alcohol consumption) for the sample of individuals aged 15 years or older, n= 25,324.

| Table S2. Survey characteristics | |  |  |  |  |  |  |
| --- | --- | --- | --- | --- | --- | --- | --- |
| Country | Number of respondents | Female | Male | % Female (unweighted) | Country income status | Year of WHO STEPs survey | WHO Region |
| Bhutan | 2,822 | 1,748 | 1,074 | 61.94 | Lower middle income | 2014 | South Asia |
| Eswatini | 3,531 | 2,303 | 1,228 | 65.22 | Lower middle income | 2014 | Southern Africa |
| Georgia | 4,212 | 2,940 | 1,272 | 69.80 | Lower middle income | 2016 | Europe |
| Guyana | 2,655 | 1,589 | 1,066 | 59.85 | Upper middle income | 2016 | Caribbean |
| Kenya | 4,488 | 2,692 | 1,796 | 59.98 | Lower middle income | 2015 | East Africa |
| Nepal | 4,143 | 2,807 | 1,336 | 67.75 | Low income | 2013 | South Asia |
| St. Vincent & the Grenadines | 3,473 | 1,941 | 1,532 | 55.89 | Upper middle income | 2013 | Caribbean |
| Total | 25,324 | 16,020 | 9,304 | 63.26 |  |  |  |
| *WHO - World Health Organization | |  |  |  |  |  |  |

Table S3. Characteristics of individuals with data on dietary behaviours, and data on waist circumference (n=23,273) hypertension (n=24,011), diabetes (n=17,724) status

|  | Population with data on waist circumference (n =23,273) | | | | Population with data on hypertension status (*n*= 24,011) | | | | Population with data on diabetes status (*n*= 17,724) | | | |
| --- | --- | --- | --- | --- | --- | --- | --- | --- | --- | --- | --- | --- |
|  | Overall | Male | Female | *p-value* | Overall | Male | Female | *p-value* | Overall | Male | Female | *p-value* |
| **Sex*** |  |  |  |  |  |  |  |  |  |  |  |  |
| Males | 50.57 (49.51, 51.64) | - | - | *-* | 49.82 (48.75, 50.88) | - | - | *-* | 47.93 (46.67, 49.31) | - | - | *-* |
| Females | 49.43 (48.36, 50.49) | - | - | *-* | 50.18 (49.12, 51.25) | - | - | *-* | 52.07 (50.69, 53.43) | - | - | *-* |
| **Age** |  |  |  |  |  |  |  |  |  |  |  |  |
| Mean age | 36.44 (36.13, 36.74) | 36.12 (35.69, 36.56) | 36.75 (36.41, 37.10) | *0.014* | 36.35 (36.05, 36.65) | 36.25 (35.82, 36.68) | 36.45 (36.10, 36.80) | *0.438* | 37.37 (36.94, 37.81) | 36.88 (36.24, 37.52) | 37.82 (37.33, 38.31) | *0.012* |
| **Educational Attainment*** |  |  |  |  |  |  |  |  |  |  |  |  |
| No formal schooling | 14.74 (13.43, 16.14) | 11.33 (11.00, 12.82) | 18.22 (16.60,19.96) | *<0.001* | 14.80 (13.49, 16.21) | 11.32 (9.99, 12.80) | 18.25 (16.63, 19.99) | *<0.001* | 14.71 (13.48, 16.03) | 11.54 (10.20, 13.04) | 17.62 (16.10, 19.24) | *<0.001* |
| Less than primary school | 9.87 (9.09, 10.71) | 10.65 (9.60, 11.80) | 9.07 (8.23, 9.99) |  | 9.85 (9.07, 10.69) | 10.62 (9.55, 11.78) | 9.09 (8.26, 10.0) |  | 10.04 (9.30, 10.82) | 10.74 (9.70, 11.89) | 9.39 (8.48, 10.39) |  |
| Primary school completed | 20.58 (19.22, 22.02) | 21.57 (19.85, 23.39) | 19.58 (18.22, 21.01) |  | 20.67 (19.30, 22.11) | 21.68 (19.96, 23.51) | 19.67 (18.31, 21.11) |  | 21.04 (19.28, 22.92) | 20.69 (18.42, 23.15) | 21.37 (19.52, 23.34) |  |
| Some secondary school | 20.63 (19.03, 22.32) | 21.03 (19.20, 22.99) | 20.21 (18.53, 22.01) |  | 20.51 (18.94, 22.18) | 20.89 (19.08, 22.83) | 20.14 (18.49, 21.89) |  | 21.17 (19.57, 22.87) | 22.08 (20.04, 24.27) | 20.34 (18.58, 22.22) |  |
| Secondary school or above | 34.18 (32.03, 36.39) | 35.41 (33.13, 37.76) | 32.92 (30.49, 35.44) |  | 34.16 (32.01, 36.38) | 35.49 (33.20,37.85) | 32.85 (30.42, 35.36) |  | 33.04 (31.17, 34.97) | 34.95 (32.56, 37.41) | 31.29 (29.15, 33.51) |  |
| **Working *** | 54.37 (52.68, 56.05) | 68.83 (67.04, 70.56) | 39.58 (37.15, 42.06) | *<0.001* | 54.08 (52.39, 55.76) | 68.74 (66.97, 70.46) | 39.54 (37.13, 41.99) | *<0.001* | 53.07 (50.82, 55.30) | 67.59 (65.23, 69.87) | 39.70 (36.78, 42.74) | *<0.001* |
| **Physical Activity** |  |  |  |  |  |  |  |  |  |  |  |  |
| Achieving 600 MET a week | 84.79 (82.93, 86.47) | 89.16 (87.88, 90.32) | 80.31 (77.56, 82.80) | *<0.001* | 84.62 (82.71, 86.35) | 88.99 (87.68, 990.18) | 80.28 (77.46, 82.84) | *<0.001* | 84.36 (82.42, 86.12) | 89.26 (87.64) | 79.85 (77.18, 82.28) | *<0.001* |
| **Alcohol consumption** |  |  |  |  |  |  |  |  |  |  |  |  |
| Mean number of drinks per week | 3.86 (3.46, 4.26) | 6.43 (5.76, 7.10) | 1.24 (1.01. 1.46) | *<0.001* | 3.81 (3.42, 4.21) | 6.43 (5.76, 7.10) | 1.22 (1.00, 1.44) | *<0.001* | 3.67 (3.24, 4.10) | 6.36 (5.58, 7.14) | 1.19 (0.96, 1.42) | *<0.001* |
| *Consuming alcohol during a week* |  |  |  |  |  |  |  |  |  |  |  |  |
| No alcohol use reported | 70.35 (68.94, 71.73) | 56.13 (54.24, 58.00) | 84.90 (83.42, 86.27) | *<0.001* | 70.67 (69.29, 72.01) | 56.16 (54.28, 58.01) | 85.06 (83.63, 86.39) | *<0.001* | 71.68 (69.92, 73.38) | 56.89 (54.47, 59.28) | 85.28 (83.57, 86.85) | *<0.001* |
| Consume one alcoholic drink or more | 29.64 (28.27, 31.06) | 43.87 (42.00, 45.76) | 15.10 (13.73, 16.58) |  | 29.33 (27.99, 30.71) | 43.84 (41.99, 45.72) | 14.94 (13.61, 16.37) |  | 28.32 (26.62, 30.81) | 43.11 (40.72, 45.53) | 14.72 (13.15, 16.43) |  |
| **Tobacco use (smoke or smokeless) *** | |  |  |  |  |  |  |  |  |  |  |  |
| No tobacco use | 69.454 (68.09, 70.79) | 51.75 (49.78, 53.71) | 87.57 (86.61, 88.47) | *<0.001* | 69.71 (68.33, 71.05) | 51.50 (49.51, 53.48) | 87.78 (86.83, 88.67) | *<0.001* | 70.90 (69.56, 72.21) | 52.02 (49.89, 54.14) | 88.29 (87.11, 89.37) | *<0.001* |
| Past use of tobacco | 19.41 (18.26, 20.61) | 31.87 (30.06. 33.73) | 6.66 (6.00, 7.39) |  | 19.26 (18.10, 20.48) | 32.10 (30.24, 34.01) | 6.52 (5.87, 7.24) |  | 17.59 (16.48, 18.77) | 30.20 (28.33, 32.13) | 5.99 (5.32, 6.74) |  |
| Current use of tobacco | 11.14 (10.45, 11.87) | 16.38 (15.24, 17.59) | 5.77 (5.21, 6.39) |  | 11.03 (10.35, 11.79) | 16.40 (15.27, 17.60) | 5.70 (5.15, 6.30) |  | 11.50 (10.54, 12.54) | 17.78 (16.02, 19.69) | 5.72 (4.96, 6.59) |  |
| **Obesity measures** |  |  |  |  |  |  |  |  |  |  |  |  |
| ***Waist circumference*** |  |  |  |  |  |  |  |  |  |  |  |  |
| Mean wasit circumference | 85.25 (84.81, 85.70) | 84.49 (84.02, 84.96) | 86.03 (85.38, 86.68) | *<0.001* | 85.21 (84.76, 85.66) | 84.48 (84.01, 84.95) | 85.96 (85.30, 86.62) | *<0.001* | 86.13 (85.50, 86.76) | 84.99 (84.34, 85.64) | 87.22 (86.38, 88.06) | *<0.001* |
| High waist circumference** | 26.11 (25.07, 27.17) | 11.15 (10.32, 12.03) | 41.42 (39.82, 43.04) | *<0.001* | 26.02 (24.99, 27.08) | 11.10 (10.28, 11.98) | 41.33 (39.72, 42.96) | *<0.001* | 28.86 (27.28, 30.50) | 12.21 (10.94, 13.61) | 44.73 (42.51, 46.97) | *<0.001* |
| **Blood pressure measures** |  |  |  |  |  |  |  |  |  |  |  |  |
| Mean systolic blood pressure | 126.00 (125.65, 126.35) | 128.33 (127.83, 128.82) | 123.61 (123.14, 124.07) | *<0.001* | 125.85 (125.50, 126.21) | 128.49 (127.99, 128.99) | 123.24 (122.78, 123.69) | *<0.001* | 127.02 (126.56, 127.48) | 129.51 (128.84, 130.19) | 124.72 (124.18, 125.26) | *<0.001* |
| Mean diastolic blood pressure | 79.87 (79.50, 80.25) | 79.87 (79.34, 80.39) | 79.88 (79.51, 80.26) | *0.945* | 79.78 (79.41, 80.15) | 79.92 (79.41, 80.43) | 79.64 (79.28, 80.00) | *0.257* | 80.46 (80.06, 80.56) | 80.71 (80.09, 81.33) | 80.23 (79.86, 80.60) | *0.136* |
| Hypertension* | 26.95 (26.05, 27.86) | 27.22 (25.88, 28.61) | 26.67 (25.69, 27.67) | *0.487* | 26.75 (25.88, 27.64) | 27.49 (26.16, 28.85) | 26.02 (25.07, 26.98) | *0.057* | 30.04 (28.77, 31.34) | 30.67 (28.75, 32.67) | 29.46 (28.15, 30.80) | *0.251* |
| Self-reported diagnosed hypertension | 11.30 (10.79, 11.83) | 8.52 (7.87, 9.21) | 14.15 (13.43, 14.90) | *<0.001* | 11.32 (10.82, 11.85) | 8.79 (8.15, 9.48) | 13.83 (13.12, 14.58) | *<0.001* | 14.23 (13.33, 15.17) | 11.00 (9.91, 12.21) | 17.19 (16.04, 18.41) | *<0.001* |
| Undiagnosed hypertension | 15.65 (14.92, 16.41) | 18.70 (17.55, 19.91) | 12.52 (11.80, 13.27) |  | 15.43 (14.71, 16.17) | 18.69 (17.54, 19.91) | 12.18 (11.49, 12.91) |  | 15.18 (14.93, 16.73) | 19.67 (18.17, 21.26) | 12.26 (11.41, 13.18) |  |
| **Blood glucose measures** |  |  |  |  |  |  |  |  |  |  |  |  |
| Blood glucose |  |  |  |  |  |  |  |  |  |  |  |  |
| Mean BGL | 4.84 (4.80, 4.88) | 4.79 (4.74, 4.84) | 4.89 (4.84, 4.95) | *0.001* | 4.83 (4.79, 4.87) | 4.79 (4.74, 4.84) | 4.87 (4.82, 4.93) | *0.008* | 4.82 (4.76, 4.89) | 4.73 (4.67, 4.80) | 4.91 (4.82, 5.00) | *<0.001* |
| Diabetes* | 5.80 (5.20, 6.45) | 4.89 (4.24, 5.62) | 6.69 (5.94, 7.54) | *<0.001* | 5.80 (5.22, 6.44) | 4.94 (4.30, 5.67) | 6.63 (5.89, 7.44) | *<0.001* | 10.02 (8.98, 11.17) | 7.59 (6.48, 8.87) | 12.26 (10.90, 13.76) | *<0.001* |
| Self-reported diagnosed diabetes | 3.37 (2.84, 3.99) | 2.55 (2.06, 3.14) | 4.18 (3.49, 5.01) | *<0.001* | 3.36 (2.84, 3.97) | 2.58 (2.10, 3.18) | 4.11 (3.43, 4.91) | *<0.001* | 7.54 (6.51, 8.71) | 5.22 (4.23, 6.43) | 9.67 (8.31, 11.21) | *<0.001* |
| Undiagnosed diabetes | 1.76 (1.50, 2.05) | 1.72 (1.35, 2.18) | 1.79 (1.51, 2.14) |  | 1.78 (1.52, 2.08) | 1.72 (1.36, 2.19) | 1.83 (1.51, 2.22) |  | 1.91 (1.61, 2.27) | 1.76 (1.33, 2.31) | 2.05 (1.67, 2.51) |  |

* Percent accounts for sampling design with survey weights re-scaled by the survey’s sample size such that all countries contribute equally to estimates, for each outcome sample.

**Definition of high waist circumference, waist ≥ 102cm for males and waist ≥ 88cm for females

Table S4. Prevalence of dietary behaviours among participants with data on waist circumference (n=23,273) hypertension (n=24,011), or diabetes (n=17,724) status

|  | Population with data on waist circumference (*n =*23,957) | | | | Population with data on hypertension status (*n*= 24,719) | | | | Population with data on diabetes status (*n*= 17,724) | | | |
| --- | --- | --- | --- | --- | --- | --- | --- | --- | --- | --- | --- | --- |
|  | Overall | Male | Female | *p-value* | Overall | Males | Females | *p-value* | Overall | Male | Female | *p-value* |
| ***Salt use behaviour*** |  |  |  |  |  |  |  |  |  |  |  |  |
| Positive salt behaviour (>50%) | 29.19 (26.64, 31.90) | 27.18 (24.59, 29.94) | 31.26 (28.46, 34.22) | *<0.001* | 29.28 (26.77, 31.92) | 27.24 (24.64, 30.01) | 31.30 (28.59, 34.13) | *<0.001* | 30.68 (27.90, 33.61) | 27.85 (24.87, 31.03) | 33.29 (30.30, 36.43) | *<0.001* |
| ***Fruit and vegetable consumption*** |  |  |  |  |  |  |  |  |  |  |  |  |
| Met WHO guidelines (400g per day) | 13.96 (12.75, 15.26) | 14.67 (13.08, 16.42) | 13.23 (12.09, 14.46) | *0.047* | 13.99 (12.79, 15.30) | 14.76 (13.18, 16.50) | 13.22 (12.09, 14.44) | *0.025* | 13.86 (12.70, 15.11) | 14.84 (13.23, 16.60) | 12.96 (11.76, 14.25) | *0.027* |
| ***Fat and oil used in cooking*** |  |  |  |  |  |  |  |  |  |  |  |  |
| Vegetable | 93.39 (92.22, 94.40) | 93.05 (91.57, 94.29) | 93.75 (92.67, 94.68) | *0.500* | 93.40 (92.21, 94.42) | 93.01 (91.53, 94.25) | 93.79 (92.67, 94.74) | *0.521* | 93.39 9(91.97, 94.59) | 92.85 (90.97, 94.36) | 93.90 (92.57, 95.00) | *0.482* |
| Animal | 2.48 (2.01, 3.06) | 2.54 (1.92, 3.35) | 2.42 (1.97, 2.97) |  | 2.48 (2.01, 3.05) | 2.57 (1.96, 3.38) | 2.38 (1.94, 2.92) |  | 2.22 (1.85, 2.69) | 2.43 (1.87, 3.16) | 2.04 (1.68, 2.48) |  |
| Other | 2.97 (2.14, 4.12) | 3.13 (2.11, 4.61) | 2.82 (2.09, 3.78) |  | 2.97 (2.13, 4.13) | 3.16 (2.13, 4.64) | 2.78 (2.04, 3.77) |  | 3.14 (2.11, 4.64) | 3.41 (2.11, 5.47) | 2.89 (2.00, 4.14) |  |
| None in particular | 0.47 (0.36, 0.62) | 0.49 (0.34, 0.72) | 0.46 (0.33, 0.63) |  | 0.47 (0.36, 0.62) | 0.49 (0.33, 0.71) | 0.45 (0.33, 0.62) |  | 0.59 (0.41, 0.84) | 0.59 9(0.34, 0.99) | 0.59 (0.40, 0.88) |  |
| None | 0.68 (0.50, 0.91) | 0.79 (0.52, 1.20) | 0.68 (0.50, 0.91) |  | 0.69 (0.51, 0.91) | 0.77 (0.51, 0.12) | 0.60 (0.44, 0.80) |  | 0.65 (0.48, 0.87) | 0.72 (0.45, 1.13) | 0.58 (0.42, 0.81) |  |

* Percent accounts for sampling design with survey weights re-scaled by the survey’s sample size such that all countries contribute equally to estimates, for each outcome sample.

Figure S1a. Weighted proportion of men (n=8,551) reporting positive dietary behaviours, in seven low-and middle-income countries

Figure S1b. Weighted proportion of women (n=14,960) reporting positive dietary behaviours, in seven low-and middle-income countries

**10.1%**

**26.6%**

**2.6%**

**0.3%**

**0.4%**

**11.6%**

**2.7%**

**22.6%**

Figure S2.b. Percentage (95% confidence interval) of women with undiagnosed hypertension, by the self-report of salt behaviour on a seven point scale*

Figure S2.a. Percentage (95% confidence interval) of men with undiagnosed hypertension, by the self-report of salt behaviour on a seven point scale*

*Model adjusted for type of fat and oil used in cooking, age, education, working status, physical activity, alcohol use, tobacco use and waist circumference

Figure S3.b. Percentage (95% confidence interval) of women with undiagnosed hypertension, by the self-report of salt behaviour on a four point scale*

Figure S3.a. Percentage (95% confidence interval) of men with undiagnosed hypertension, by the self-report of salt behaviour on a four point scale*

*Model adjusted for type of fat and oil used in cooking, age, education, working status, physical activity, alcohol use, tobacco use and waist circumference
